# Infinitessimalism Again

**Published:** 1884-01

**Authors:** C. S. Carr


### INFINITESIMALISM AGAIN.

C. S. CARR, M. D.

The adherents of infinitesimalism use remedies (so called) which they believe to possess peculiar virtues, acquired by the process of dilution or trituration, from the 30th attenuation to the 5,000th, and he who elects himself as an apologist or defender of the system, has taken upon himself the task of showing, either, that in the attenuations above named there is the actual presence of the drug, or that the drug has imparted to the attenuating material virtues which it did not originally possess.

Upon the latter supposition we have nothing to say at present, but that any virtue can be justly attributed to the actual presence of the drug in any attenuation above the 15th, we must insist is unscientific, and he who practices it, or defends it, must do so in utter defiance of undoubted facts, furnished by modern chemistry.

We propose in this paper to deal with nothing but *facts* and *figures*, and leave critics to choose between the two, which they will oppose. Of course it is admitted that the size, number, and weight of molecules, as ascertained by modern chemistry are but approximations. The fact that they are approximations does not invalidate their testimony any more than the fact that astronomical distances being approximations, are to be rejected, and are not to be cited to disprove the alleged "experience" of biased observers.

At the risk of being prolix we will not abridge the calculation for fear of being misunderstood.

The first *fact* of modern chemistry which we cite is that the actual weight of a molecule of hydrogen is one hundred quintillionth of a milligram; that is to say, two hundred quintillion molecules of hydrogen weigh one milligram.

Second—The relative weight of hydrogen and sulphur molecules are as 1 to 32, the sul pher molecule being 32 times as heavy as the hydrogen molecule. Therefore it would take as many molecules of sulphur to weigh one milligram as 32 is contained times in two hundred quintillion, or six quintillion two hundred and fifty quadrillion.

Now we propose to take our milligram of sulphur and subject it to the first centisimal trituration, which would give us in each milligram of the trituration as many molecules of sulphur as one hundred is contained times in six quintillion two hundred and fifty quadrillion, or sixty-two quadrillion five hundred trillion.

Taking one milligram of our first trituration we subject it to the second trituration, and each milligram will contain one hundred times less, or six hundred and twenty-five trillions of sulphur molecules. Each milligram of the third would contain six trillion two hundred and fifty billion. A similar quantity of the fourth would contain sixty-two billion, five hundred million sulphur molecules, and the fifth would contain six hundred and twenty-five million. In the sixth trituration we would have six million two hundred and fifty thousand, while the seventh would give us sixty-two thousand and five hundred. The eight will turn out just six hundred and twenty-five molecules, and one milligram of the ninth trituration will be entitled to six and a quarter molecules. The tenth trituration would only allow us one molecule to sixteen milligrams of the trictura-tion, and of the eleventh we should be obliged to have sixteen hundred milligrams to be entitled to a single molecule. Now sixteen hundred milligrams are equal to a little over twenty-four grains, or one-eighteenth of a pound avoirdupois, nearly. Of the twelfth trituration we would have one molecule of sulphur to a trifle less than six pounds.

It would take six hundred pounds of triturating material to properly represent one molecule of the 13th trituration, and six hundred pounds of the 13th would have to be given at a single dose to insure the administration of a single molecule. Sixty thousand pounds would sufficiently attenuate the unfortunate molecule to entitle it to rank as the 14th POTENCY, but to finally reach the 15th we would have to furnish each molocule six million pounds of the triturating material. This is no fancy sketch, but on the contrary is a very close approximate, the truth of which must force itself upon every candid reader. Now in all soberness, with no wish to give offence to any one, let me ask what is the probability that the 30th attenuation of any drug contains any of the drug. The 30th may be called the unit of the high potency advocate, below it they scarcely ever use any remedy, while above it they soar until they are safely beyond the reach of all earthly notation 50,000th potency! just think of it. Imagine if you can a fraction whose numerator is one, and denominator is one, followed by one hundred thousand ciphers.

The almost infinite divisibility of matter has been the fortress behind which the advocate of high potency has crouched many years. It has been their pet argument in controversy, it has been their shield when the arrows of logic have fallen thick about them, but I fear it, too, will become antiquated when the light of the nineteenth century has chased back the shadows of by-gone superstitions, to the original *nothing* which begat them. Suppose, for the sake of argument, that the careful, patient estimate of the actual weight of the molecule, as made by our best scientists is too great, suppose for every one molecule of the estimate there are actually one hundred, it still remains true that our globe itself could not furnish sufficient material to raise one single molecule to the 30th; suppose for every one estimated there are actually one thousand, still a sufficient quantity of the 30th to be entitled to one molecule could not be contained within the orbit of the moon. Suppose for every one molecule of the estimate there are really one hundred million million molecules, yet it remains true that all the triturating sugar on earth, together with all that ever was on earth, would not be sufficient to represent the least amount that would be entitled to one molecule of the drug.

It would seem, however, that the estimate which forms the premise of this paper, had sufficiently recognized the extreme divisibility of matter, and there can be no doubt as to the probable accuracy of the estimate. Let us see if we can comprehend the estimate, for the divisibility of matter is almost beyond comprehension. First let us suppose we have before us a grain of sulphur, which would be a piece about as large as a good sized wheat kernel. Now suppose we divide this into two hundred pieces, each piece would hardly be visible to the unaided eye. Three of these tiny masses would weigh nearly a milligram and four of them would weigh more than a milligram, hence a milligram of sulphur is a particle so small that it requires the best of eyesight to see it at all. Now the estimate allows that each of these tiny masses contains six quintillions two hundred and fifty quadrillion molecules of sulphur. If our little mass of sulphur should lose by evaporation one million molecules each second of time, it would not have had time to lose half of its molecules since the Adamic creation. And yet figures prove that the 15th attenuation of sulphur will not have a single molecule in any less than six million pounds of triturating material. If the Infinitesimalist will insist upon the efficacy of the 30th, he must do so with the fact that it does not contain any of the drug staring him in the face, unless, like the ostrich, he hides his head in the sand of his own ignorance or bigotry, and imagine because he hides from the truth himself that others cannot see his folly.

I quote a short paragraph from the United States Medical Investigator, not because it contains any unusual statement, but because it is a typical of the teachings of infinitesimalism. Vol. V, page 499, the writer says: "When I once carefully select a remedy for a case and I apply say the 200th, and get no effect, I *never change my remedy* but go *higher* and *higher* even to the 10,000,000th potency" (provided always the patient does not die before). Is it this kind of "experience" that we are to accept and throw away our chemistries? Must we pitch our arithmetics overboard for the sake of believing such stuff, simply because somebody chooses te denominate it as his "experience?"

Nay, verily such nonsense belongs to the dead past, and it is to be hoped it will soon cease to live to cause the shame homœopathy, that grand old system of medicine to which the world owes so much, but to which infinitesimalism has clung like a fungus, causing it more stigma and just criticisms than all other causes combined.

